# Celocentesis in Ultra-Early Prenatal Diagnosis: Diagnostic Accuracy, Safety Profile, and Emerging Therapeutic Perspectives

**DOI:** 10.3390/genes17070746

**Published:** 2026-06-29

**Authors:** Stylianos Makrydimas, Efthalia Moustakli, Nektaria Zagorianakou, Emmanouil D. Oikonomou, Ioannis Mitrogiannis, George Makrydimas

**Affiliations:** 1 Medical School, Aristotle University of Thessaloniki, 54124 Thessaloniki, Greece; smakrydimas@gmail.com; 2 Department of Nursing, School of Health Sciences, University of Ioannina, 45500 Ioannina, Greece; ef.moustakli@uoi.gr; 3 Scientific Laboratory for Innovative Technologies in Internal Medicine, Preventive Medicine and Overall Care, Department of Nursing, School of Health Sciences, University of Ioannina, 45500 Ioannina, Greece; zagorianakou@uoi.gr; 4 Human Computer Interaction Laboratory, Department of Informatics and Telecommunications, University of Ioannina, Kostakioi, 47150 Arta, Greece; e.oikonomou@uoi.gr; 5 Harris Birthright Research Centre for Fetal Medicine, King’s College London, 16-20 Windsor Walk, London SE5 8BB, UK; ioannismitrogiannisgr@gmail.com; 6 Department of Obstetrics & Gynecology, University Hospital of Ioannina, 45500 Ioannina, Greece

**Keywords:** coelomic fluid, fetal cell isolation, maternal cell contamination, embryonic genetic testing, monogenic disorders, quantitative fluorescent PCR, intrauterine therapy

## Abstract

Celocentesis represents a novel form of invasive pregnancy test that allows the genetic material of the embryo to be tested during the embryonic stage at 6–9 weeks of gestation. The purpose of this narrative review is to present the latest available literature on celocentesis, including its biological basis, technical aspects, diagnostic performance, safety profile, clinical applications, and future perspectives. Available evidence from selected studies conducted in highly specialized centers suggests that the diagnosis of monogenic diseases by celocentesis can achieve high accuracy, with reported success rates ranging from 93% to 99% when combined with molecular testing and selective fetal cell isolation. Similarly, a high level of concordance with conventional prenatal and postnatal diagnostic methods has been reported. The pregnancy loss associated with celocentesis appears to be low and comparable to baseline early pregnancy loss, although current evidence is derived primarily from observational studies and limited clinical series. One of the main benefits of celocentesis is the capability to perform prenatal diagnosis at an early stage of pregnancy, which facilitates more informed decisions about treatment options, minimizes parental anxiety, and allows earlier intervention when required. Moreover, experimental evidence suggests that celocentesis may provide a future platform for intrauterine therapeutic approaches, including stem cells and gene-based therapies, although these applications remain investigational. Despite these promising findings, celocentesis should currently be considered an experimental procedure, as its use remains largely confined to specialized centers and further multicenter studies are required to establish its safety, reproducibility, and broader clinical utility.

## 1. Introduction

Prenatal diagnosis plays a vital role in today’s pregnancy management, particularly in cases at increased risk of genetic and chromosomal disorders. Diagnostic efficacy has improved with advancements in both fetal imaging and molecular genetics; however, performing tests at a particular stage in gestation presents challenges [1,2,3].

Conventional invasive techniques, including amniocentesis and chorionic villus sampling (CVS), are typically performed at approximately 15 and 11 weeks of gestation, respectively. Although these techniques are reliable and accurate, their later gestational timing limits early clinical decision-making and limits opportunities for less invasive management strategies [4,5]. Additionally, invasive operations performed earlier in pregnancy increase the risk of congenital defects and miscarriages [6].

In prenatal medicine, a genetic diagnosis in the first several weeks of pregnancy is still crucial. Early prenatal diagnosis may reduce emotional stress related to diagnostic ambiguity in addition to providing a chance to apply a less invasive treatment and boost the likelihood of successful reproduction [7,8]. Although non-invasive prenatal testing (NIPT) has advanced in detecting common aneuploidies, it remains inadequate for diagnosing monogenic diseases, underscoring the need for efficient invasive diagnostic approaches [9,10].

One approach that facilitates ultra-early prenatal diagnosis is celocentesis. This method uses ultrasound-assisted aspiration of the coelomic cavity at 6–9 weeks after conception, when it is the largest extraembryonic cavity [11,12,13].

Cells obtained from the coelomic fluid include those of embryonic origin, mainly erythroid precursors derived from the yolk sac. Therefore, fetal DNA can be reliably analyzed via this approach. This provides the benefit of conducting prenatal diagnosis at an earlier stage than other available techniques [14,15,16].

However, early studies revealed certain shortcomings in the sensitivity of celocentesis, chiefly attributed to a low number of fetal cells and a high level of maternal cell contamination (MCC) [13]. Great strides have been made toward enhancing sensitivity in this technique by developing more sensitive molecular technologies, such as PCR, quantitative fluorescent PCR (QF-PCR), and cell enrichment techniques like immunomagnetic separation and micromanipulation [17].

Recent studies have shown that celocentesis achieves a high level of concordance with standard prenatal and postnatal diagnostic procedures [13]. Apart from being an effective diagnostic method, the celocentesis technique has been offered as a means for therapeutic procedures performed directly on the fetus, as the embryo develops at a very early stage, and the fetal immunity is still underdeveloped [11,12].

However, despite numerous achievements, celocentesis remains largely confined to specialized centers and is still considered an experimental procedure. Its broader clinical adoption is limited by technical complexity, operator dependence, the lack of standardized protocols, and the scarcity of large multicenter studies evaluating its safety, reproducibility, and diagnostic reliability. The current manuscript provides a detailed overview of celocentesis, covering its biological basis, technical aspects, diagnostic accuracy, safety profile, clinical applications, limitations, and future perspectives. Compared with previous reviews, this manuscript aims to provide an updated and clinically oriented synthesis of celocentesis by integrating embryological rationale, procedural workflow, diagnostic performance, safety evidence, current implementation barriers, and emerging therapeutic perspectives within a single framework. In addition, it provides a structured comparison with established prenatal diagnostic approaches and discusses the current limitations that restrict broader clinical translation.

## 2. Literature Search Strategy

A non-systematic literature review was conducted to identify relevant studies on celocentesis and its applications in early prenatal diagnosis. Literature searches were conducted in electronic bibliographic databases, such as PubMed, Scopus, and Web of Science, for publications up to February 2026.

The literature search incorporated the following keywords and MeSH terms: “celocentesis,” “coelocentesis,” “coelomic fluid,” “early prenatal diagnosis,” “embryonic genetic testing,” and “fetal cells.” Also, the literature references in selected scientific articles were manually studied for additional sources.

Studies considered in this review include only original articles, clinical research, and experimental studies regarding the biology, technology, performance characteristics, safety, and therapeutic possibilities of celocentesis. In addition, reference lists of relevant articles were screened to identify additional sources.

Since this review was narrative in nature, no formal inclusion or exclusion criteria were established, and neither a quantitative synthesis nor a meta-analysis was carried out. The selection of studies was based on their methodological quality, usefulness, and addition to the current understanding of celocentesis.

Although no formal study selection process was performed, priority was given to original clinical studies, methodological investigations, and recent reviews reporting diagnostic performance, maternal cell contamination, safety outcomes, and emerging clinical or therapeutic applications of celocentesis. Small case series and case reports were included when they provided unique information regarding rare monogenic disorders, chromosomal abnormalities, or novel technical developments.

## 3. Embryological and Biological Basis of Celocentesis

During the early phases of human development, celocentesis allows access to material obtained from the embryo inside the coelomic cavity [13,15,18]. The largest compartment of the gestational sac between weeks six and nine of pregnancy is the coelomic cavity, which begins to form around the fifth week of pregnancy as an extraembryonic area within the extraembryonic mesoderm before being gradually destroyed by the growing amniotic cavity [19,20,21].

**Figure 1:**
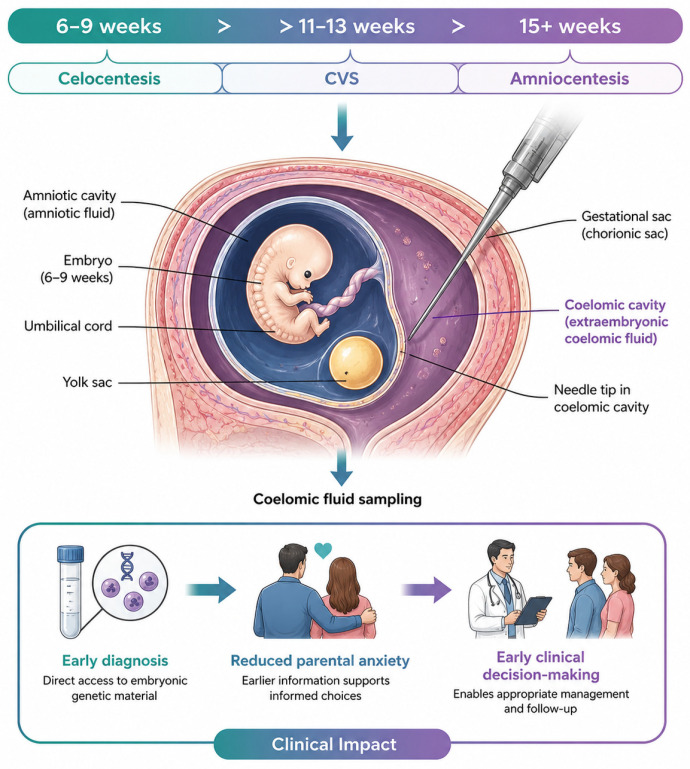
Schematic representation of celocentesis and its clinical significance. The illustration depicts the anatomical relationships between the embryo, amniotic cavity, yolk sac, chorionic (gestational) sac, and extraembryonic coelomic cavity during early pregnancy (6–9 weeks of gestation). Under ultrasound guidance, a needle is introduced into the coelomic cavity while avoiding direct puncture of the embryo and amniotic sac, allowing aspiration of coelomic fluid containing embryo-fetal cells for genetic analysis. Compared with chorionic villus sampling (11–13 weeks) and amniocentesis (≥15 weeks), celocentesis enables ultra-early prenatal diagnosis, potentially facilitating earlier clinical decision-making and reducing parental anxiety. As the procedure is technically demanding and operator-dependent, careful ultrasound guidance is essential to minimize procedural risks.

A schematic representation of the anatomical relationships and temporal window relevant to celocentesis is provided in Figure 1.

A dynamic biological environment that reflects the contributions of both the mother and the embryo is represented by coelomic fluid. The latter is defined as an ultrafiltrate of maternal blood, enriched with various growth factors, metabolites, placental proteins, and embryonic cells [22,23,24]. The yolk sac, which is the primary site of hematopoiesis during early embryogenesis, produces embryo–fetal erythroid precursor cells that are present within this cellular population. These nucleated erythroblasts constitute a key biological source for early prenatal diagnostics and provide fetal DNA for molecular analysis [25,26,27].

Prior to the gestational window in which amniocentesis and chorionic villus sampling can be safely performed, the presence of fetal cells in the coelomic cavity offers a unique opportunity for ultra-early prenatal diagnosis [28,29]. However, the biological composition of coelomic fluid poses a significant challenge due to the presence of predominantly epithelial maternal cells [22,30].

The presence of maternal cells has often posed problems to the process of celocentesis, as it has been one of the key reasons behind its high failure rate. The failure rate reached up to 40%, and, together with the low concentration of fetal cells, limits the clinical use of the procedure [12,13].

Techniques for diagnosing cell and molecular biology have seen many improvements, making the process of celocentesis more accurate [13]. The effective isolation of the cells from the embryo-fetus may be attained via selective techniques that include immunomagnetic sorting using particular surface marker proteins (CD71-positive erythroid cells) [31,32]. In addition, cell isolation may be accomplished through micromanipulation techniques based on morphological criteria, even in the presence of MCC [16,33].

Advances in molecular diagnostics, driven by the development of PCR and QF-PCR assays, have enhanced sensitivity. This enables analysis of genetic material from a small number of isolated fetal cells, along with small quantities of DNA. In this regard, celocentesis has progressed from an impractical method to a realistic one [33,34,35].

The physiological features of the coelomic cavity may facilitate its future use in therapeutic therapies in addition to its diagnostic function. The immunological immaturity of early fetal development may facilitate the engraftment of transplanted cells [36,37,38,39]. Celocentesis may therefore be a feasible method for intrauterine gene or cell therapy. Experiments on non-human primates have shown that human hematopoietic stem cells may survive in coelomic fluid and that aspirating and refilling fluid is safe [12].

The diagnostic and potentially therapeutic applications of celocentesis in prenatal medicine are based on the unique temporal and physiological characteristics of coelomic cavity development [13].

## 4. Technique and Procedural Considerations

**Figure 2:**
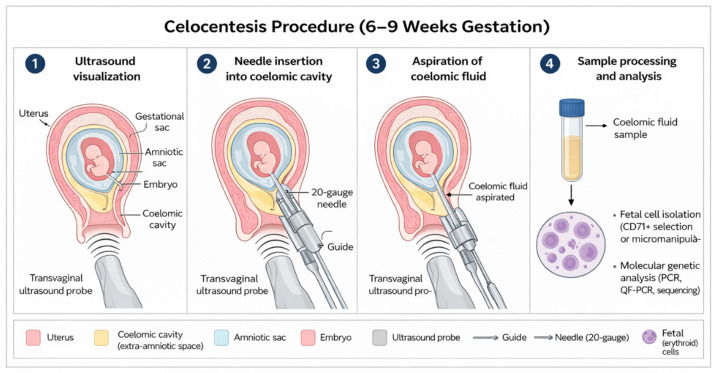
Schematic representation of the celocentesis procedure. During early pregnancy (6–9 weeks of gestation), a fine needle (20-gauge) is introduced under transvaginal ultrasound guidance into the extraembryonic coelomic cavity, while avoiding the embryo and amniotic sac within the chorionic (gestational) sac. Aspirated coelomic fluid is subsequently processed for fetal cell isolation and molecular genetic analysis. Careful ultrasound guidance is required to minimize procedural risks and maternal cell contamination.

During the early stages of pregnancy, typically six to ten weeks after conception, the coelomic fluid can be extracted using an invasive technique called celocentesis that uses ultrasound technology (celocentesis: current evidence and future directions). With a fine (20-gauge) needle, the treatment is most frequently carried out transvaginally, providing direct access to the coelomic cavity under ultrasound guidance. Figure 2 shows the stages involved in celocentesis [11,12].

After the gestational sac, amniotic cavity, and coelomic cavity have differentiated, the needle enters the extra-amniotic area. One to two milliliters of fluid are aspirated from the coelomic space [21]. To minimize maternal cell contamination (MCC), the first part of aspirated coelomic fluid is usually removed since it can contain maternal blood or decidual cells [40,41,42].

The patient tolerates this procedure really well, it causes only minor discomfort, and general anesthesia is not necessary. The majority of treatments are finished quickly, and real-time ultrasound guidance improves procedural accuracy and safety [43].

### 4.1. Sample Processing and Laboratory Workflow

Accurate diagnosis with celocentesis depends on the collected material being properly processed [13]. Since there are usually only a few fetal cells in the coelomic fluid, which are sometimes combined with maternal cells, the detection and isolation of fetal cells require specific methods [33,44,45].

The initial step in sample preparation is centrifugation, which is followed by microscopic analysis. The morphological characteristics of the embryo-fetal cells, such as their spherical shape and the nucleus’s eccentric location, are used to identify them [16,33]. They are similar to yolk sac-derived erythroid precursor cells. When there is substantial MCC, more sophisticated cell selection methods are used [46,47].

To treat MCC, two primary strategies have been put forth. Using certain surface markers, such as CD71, fetal erythroid precursor cells are separated by immunomagnetic selection in the first method. The second strategy uses micromanipulation techniques that allow for the unique isolation of each fetal cell [48,49].

After cell separation, genetic analysis is performed using extremely sensitive molecular techniques such as PCR, QF-PCR, and sequencing. It enables accurate diagnosis of monogenic disorders from minimal DNA derived from a small number of isolated cells [31,50,51,52].

### 4.2. Technical Challenges and Pitfalls

Currently, the procedure remains highly challenging, requiring further optimization in light of certain limitations. Firstly, samples, especially those containing little fetal material, could be influenced by MCC [40,53,54]. Additionally, the small volume of aspirated fluid, together with the low number of fetal cells, requires complex laboratory methods [42,55].

Another key limitation is operator proficiency, which strongly influences the effectiveness of the procedure. Moreover, differences in laboratory procedures across institutions lead to variability in test results [56].

Nevertheless, due to recent developments in ultrasound technology, cell separation procedures, and molecular diagnostics, the technique has become quite applicable. Therefore, celocentesis has the potential for early diagnosis of disorders [15].

## 5. Diagnostic Accuracy and Molecular Applications

### 5.1. Diagnostic Performance

Advances in molecular genetics and fetal cell enrichment procedures have significantly improved test accuracy. Early studies reported variable accuracy rates (58–95%) due to issues in sample preparation [33,57,58].

Currently, data from experienced specialists indicate high performance, with accuracy rates ranging from 93% to 99%. In addition, the reliability of celocentesis has been proven through a high degree of concordance with conventional methods for prenatal diagnostics, including amniocentesis, chorionic villus sampling, and postnatal testing [11,12,13].

### 5.2. Applications in Monogenic Disorders

Celocentesis is also widely utilized in diagnosing monogenic diseases at an early stage during pregnancy. β-thalassemia and Hb Lepore syndromes can be accurately diagnosed starting from 7 to 9 weeks after conception [59,60].

It enables the diagnosis of various inherited diseases depending on parental mutations. The following is a list of other diseases that can be detected using this method: cystic fibrosis and a rare disease called Cockayne syndrome [61,62,63].

### 5.3. Molecular Detection of Common Aneuploidies

In addition to the detection of monogenic disorders, celocentesis can be applied to identify chromosomal abnormalities using molecular techniques such as QF-PCR. This method enables the diagnosis of sex chromosome disorders, as well as common aneuploidies involving chromosomes 13, 18, and 21 [64,65]. It should be noted that, during very early gestation, chromosomal abnormalities associated with early embryonic loss, including triploidy and other autosomal trisomies, may be encountered more frequently than at later stages of pregnancy due to the natural selection of affected embryos throughout gestation.

### 5.4. Diagnostic Limitations and Future Genetic Applications

The efficacy of fetal cell enrichment and specimen quality, especially in early gestational samples where cellularity tends to be poor, are important considerations that impact the performance of celocentesis [66,67]. An additional limitation is the potential impact of embryonic mosaicism on diagnostic interpretation. Because genetic analyses may be performed on a limited number of isolated fetal cells, or in some cases single cells, there is a theoretical risk that the sampled cells may not fully represent the genetic constitution of the developing embryo. Despite its high diagnostic accuracy, the widespread clinical implementation of celocentesis has been hampered by a lack of standardization, limited availability outside specialized centers, and the scarcity of large prospective multicenter studies confirming its safety, reproducibility, and diagnostic reliability across different clinical settings. The main clinical and methodological studies that form the current evidence base for celocentesis are summarized in Table 1.

## 6. Safety and Complications

### 6.1. Procedure-Related Pregnancy Loss

Since its inception, celocentesis’s safety profile has been a major source of worry, mainly because of the procedure’s early gestational age [68]. Initially, there was concern about its therapeutic application because early trials revealed varying rates of fetal loss [69,70].

According to more current prospective data, the procedure-related risk of miscarriage is comparable to baseline early pregnancy loss and is comparatively low [6,71,72]. In a large cohort study, most continuing pregnancies ended in live deliveries, with a miscarriage rate of about 2.3% after celocentesis. With estimated procedure-related risks of about 2%, previous comparative trials with control groups found no statistically significant increase in fetal loss [6,73].

When considered collectively, these results suggest that celocentesis does not seem to significantly raise the risk of pregnancy loss above expected baseline rates in early gestation when carried out by skilled operators [13,68].

### 6.2. Maternal Safety and Procedural Tolerability

Celocentesis is typically performed without general anesthesia and is well tolerated. Only a small percentage of patients report significant discomfort during the surgery, whereas the majority report no or only mild discomfort [13].

The research that is now available does not regularly report any serious maternal problems, such as bleeding or infection. The procedure’s favorable safety profile is a result of its minimally invasive nature and real-time ultrasound guidance [74,75].

### 6.3. Technical and Biological Risk Factors

Despite its overall safety, procedural risk may be influenced by several factors. Since precise needle placement within the coelomic cavity is necessary to prevent harm to the embryo or disruption of the amniotic sac, operator experience is still a crucial factor [76,77].

Additionally, there are intrinsic technological difficulties in the early gestational environment. Compared to later invasive procedures, the tiny size of embryonic structures and the dynamic nature of early pregnancy enhance procedural complexity [78,79].

MCC primarily represents a diagnostic limitation but may also indicate technical difficulty during the procedure and suboptimal sampling [41,42].

### 6.4. Evidence from Experimental and Preclinical Studies

Further information about the safety of celocentesis and its potential for future therapeutic applications is provided by experimental research conducted in non-human primate models. These studies demonstrate that coelomic fluid aspiration and replacement can be performed without adverse effects on embryonic development, with normal fetal and neonatal outcomes [13,80].

Additionally, human hematopoietic stem cells have been shown to remain viable within coelomic fluid, indicating biological compatibility of the coelomic environment for potential intrauterine interventions. These results imply that celocentesis may be a viable option for early treatment administration in addition to being a safe diagnostic technique [14,81].

### 6.5. Limitations of Current Safety Evidence

There is still little data on the safety of celocentesis, despite promising results. The majority of studies have very modest sample sizes and come from specialist centers with extensive procedural experience [13,68]. In addition, the lack of well-designed, large-scale randomized trials and long-term follow-up data precludes definitive conclusions regarding rare or long-term complications [82,83].

Therefore, despite encouraging safety data, celocentesis should still be considered a developing technique that requires further validation [13].

## 7. Clinical Utility and Positioning in Prenatal Diagnosis

### 7.1. Indications and Target Population

Pregnancies at increased risk of inherited monogenic disorders represent the primary indication for celocentesis, particularly when the parental pathogenic variants are known [84]. Early genetic diagnosis is essential for informed clinical decision-making in conditions such as hemoglobinopathies, cystic fibrosis, and other severe autosomal recessive disorders [85,86].

Celocentesis may also be considered in situations where early fetal genetic assessment is required, such as in pregnancies with a strong family history or a history of previously affected offspring. However, its application in low-risk populations remains limited [12,60].

### 7.2. Clinical Advantages of Ultra-Early Diagnosis

The main clinical benefit of this approach is its ability to detect disease weeks earlier than standard methods, during the embryonic phase of pregnancy. This time difference can have profound implications for treatment [12,87].

In normal pregnancies, early diagnosis will help reduce the parents’ stress levels and offer reassurance. In abnormal cases, early termination, usually done before the 10th week of gestation, can be considered easier and less psychologically challenging than later abortion procedures [88,89].

Finally, early detection may enable prenatal treatment, as the fetal immune system is not yet fully developed [90].

### 7.3. Comparison with Conventional Techniques

Celocentesis should be considered within the broader context of reproductive genetic testing strategies. In addition to conventional prenatal diagnostic procedures such as chorionic villus sampling (CVS) and amniocentesis, preimplantation genetic testing (PGT) represents an alternative approach for couples at risk of transmitting genetic disorders. Unlike prenatal diagnostic techniques, PGT is performed in conjunction with in vitro fertilization prior to embryo transfer, allowing the selection of unaffected embryos before pregnancy is established. However, its application is limited to couples undergoing assisted reproductive treatment. A quantitative comparison of the diagnostic characteristics, procedural requirements, clinical maturity, and reported pregnancy loss rates associated with celocentesis, CVS, amniocentesis, and PGT is presented in Table 2.

### 7.4. Current Clinical Limitations and Implementation Constraints

Although celocentesis has several potential clinical applications, its use remains largely confined to specialized centers due to its technical complexity and operator dependence [13]. In addition, maternal cell contamination necessitates advanced techniques for fetal cell isolation, further limiting its broader clinical applicability [9,107]. Moreover, the lack of standardized protocols and the limited availability of large prospective multicenter studies continue to hinder wider clinical adoption and underscore the need for further validation of the procedure’s safety, reproducibility, and diagnostic performance. Consequently, the procedure is currently available only in a limited number of highly specialized centers with the required clinical and laboratory expertise.

### 7.5. Future Role in Clinical Practice

As technological and methodological advances continue, celocentesis may become a useful tool for prenatal diagnosis in selected clinical settings. Its broader clinical application will depend on further standardization of the procedure, improvements in fetal cell isolation techniques, and increased operator experience [12,60,87].

In addition to its diagnostic role, celocentesis enables very early detection of monogenic diseases in selected high-risk pregnancies [13,15]. However, before this technique can be routinely implemented in clinical practice, its diagnostic advantages must be carefully weighed against its technical limitations and procedural challenges [108,109].

The use of celocentesis also raises important ethical and organizational considerations. Because testing is performed during the embryonic stage, appropriate genetic counseling and informed consent are essential to support informed decision-making and to address the potential implications of early diagnostic findings. Particular attention should be given to the interpretation of uncertain results, including mosaic findings or variants of uncertain significance, which may complicate clinical decision-making at a very early stage of pregnancy and increase parental anxiety. Furthermore, the limited availability of specialized centers, the need for operator expertise, and the absence of standardized clinical protocols may restrict equitable access to the procedure. At present, celocentesis should be regarded as an investigational technique and its use should remain confined to specialized centers with appropriate clinical governance and ethical oversight until further multicenter validation becomes available.

## 8. Future Perspectives

### 8.1. Technological Advances and Standardization

Despite improvements, the adoption of this technique is hindered by a lack of standardized procedures and variability in the technology used [110,111]. Further research should aim to improve the reproducibility of this procedure by introducing better methods for obtaining fetal cells, standardized sampling approaches, and molecular diagnostics [33,112,113].

Technologies that can provide analysis of genomic data using small amounts of DNA, such as digital PCR and NGS, may improve the effectiveness of this procedure [114]. Finally, advances in ultrasound imaging and biopsy guidance may help mitigate procedural risks. Importantly, large prospective multicenter studies are needed to further establish the safety, reproducibility, and diagnostic reliability of celocentesis and to facilitate the development of standardized clinical protocols for broader implementation [115,116].

### 8.2. Expansion of Diagnostic Applications

Celocentesis may also be used to diagnose diseases other than monogenic disorders [15]. Genome-wide analysis, including the detection of copy number variants and chromosomal abnormalities during early embryo development, may become feasible with advances in molecular biology [117,118].

Moreover, when combined with other diagnostic approaches, including the analysis of placental cell-free DNA through non-invasive prenatal testing (NIPT), celocentesis may contribute to a more comprehensive prenatal diagnostic framework [119]. However, unlike celocentesis, NIPT primarily reflects placental rather than direct fetal genetic material and may therefore be affected by biological factors such as confined placental mosaicism. NIPT is widely used in clinical practice because it is non-invasive, scalable, and demonstrates high sensitivity and specificity for common fetal aneuploidies. Nevertheless, it remains a screening rather than a diagnostic test and may have limitations in the detection of certain monogenic disorders and complex genomic abnormalities [9,10]. In contrast, celocentesis provides direct access to embryo-fetal cells at an earlier gestational stage and may offer definitive molecular diagnosis in selected high-risk pregnancies, although its invasive nature, technical complexity, and limited availability currently restrict its broader clinical applicability.

### 8.3. Therapeutic Potential of Celocentesis

While celocentesis is currently used for diagnostic purposes, its access to the early gestational environment has prompted interest in potential future therapeutic applications. This is supported by the relative immunological immaturity of the early fetus, which may favor engraftment of transplanted cells; however, such applications remain experimental [120].

Experimental studies in animal models have shown that human hematopoietic stem cells can survive in the coelomic environment, and that partial aspiration and replacement of coelomic fluid may be feasible without immediate adverse effects on embryonic development [81,121]. However, the use of celocentesis as a route for stem cell transplantation or gene therapy remains experimental and has not yet been established in clinical practice [122,123].

Certain monogenic conditions, such as hemoglobinopathies and cystic fibrosis, may benefit greatly from intervention from the embryonic stage, since it may alter the course of the condition [124,125]. Optimizing delivery strategies, ensuring consistent engraftment, reducing off-target effects, and addressing ethical issues are some of the major obstacles [126,127]. The possible uses are listed in Table 2.

Celocentesis may provide a special platform for early treatment intervention, especially in the context of monogenic conditions, as shown in Table 3. Nevertheless, most proposed applications remain at a preclinical or theoretical stage, highlighting the need for further experimental and clinical research.

### 8.4. Integration into Future Fetal Medicine

Celocentesis is a procedure that can transform prenatal diagnosis in the emerging field of fetal medicine by providing fetal genetic material at very early stages of pregnancy [13].

This technology can be used in combination with more advanced clinical programs that integrate early genetic testing with intrauterine therapy when necessary [139]. Validation of this method’s effectiveness requires clinical studies, continued technological development, and consideration of ethical implications. Although celocentesis shows considerable potential, its future role in prenatal care will depend on further clinical validation, standardization, and demonstration of safety and reproducibility across diverse clinical settings [140,141,142].

However, the current evidence base is subject to several limitations. Most published studies originate from a small number of highly specialized centers and involve highly selected high-risk populations, which may limit the generalizability of the reported findings. Furthermore, the available literature is largely based on observational studies and case series, while large prospective multicenter studies and randomized comparisons are lacking. Variability in operator expertise, laboratory protocols, and fetal cell isolation techniques may also influence diagnostic performance and reported safety outcomes.

## 9. Limitations

Despite its potential advantages, celocentesis has several important limitations that currently restrict its widespread clinical use [13]. The available literature is largely based on single-center studies, and although the reported results are promising, there is a lack of prospective multicenter trials to support robust conclusions regarding the safety, reproducibility, and diagnostic reliability of the procedure across different clinical settings [143]. In addition, variability in patient selection and procedural techniques further complicates the interpretation and generalization of findings. A key limitation is the absence of standardized protocols for both sampling and laboratory analysis [144].

MCC remains a major technical challenge. The coexistence of maternal and fetal cells in coelomic fluid, particularly in cases with low fetal cell counts, may compromise diagnostic accuracy [40,145]. Although advances in cell isolation and molecular techniques have improved detection, MCC continues to represent a significant obstacle [146].

Celocentesis is a highly specialized and technically demanding procedure that requires considerable operator expertise, particularly given the small size of embryonic structures and the rapid developmental changes occurring at early gestational stages. As a result, its use is currently limited to specialized centers [13].

At present, celocentesis is primarily applied to the early molecular diagnosis of genetic disorders in high-risk pregnancies [59]. However, there is insufficient evidence to support its broader application, including chromosomal analysis and genome-wide screening, particularly in low-risk populations [147,148,149].

Furthermore, the long-term effects of celocentesis on pregnancy outcomes and postnatal health have not been adequately investigated. While short-term data are generally reassuring, the absence of long-term follow-up limits definitive conclusions regarding safety [13].

Finally, although celocentesis has been proposed as a potential route for intrauterine therapeutic interventions, including gene and stem cell delivery, current evidence remains limited and largely experimental [12].

In addition to the limitations of the technique itself, the available evidence base has important methodological constraints. Most published studies originate from a small number of highly specialized centers and involve relatively small sample sizes and highly selected high-risk populations. Furthermore, differences in laboratory protocols, fetal cell isolation techniques, and outcome reporting contribute to heterogeneity across studies. As this manuscript was designed as a narrative review, no formal study quality assessment or quantitative synthesis was performed, and publication bias cannot be excluded.

## 10. Conclusions

The procedure of celocentesis provides a means by which embryonic genetic material can be accessed at the stage between 6 to 9 weeks of gestation. Available evidence from specialized centers suggests that advances in fetal cell isolation and molecular diagnostic techniques have substantially improved the diagnostic performance of celocentesis in selected clinical settings. The main strength of celocentesis is the opportunity to make a diagnosis at the embryonic stage and thus help to make early decisions while reducing stress from later-stage procedures.

While these advantages highlight the potential of celocentesis, the procedure remains a technically demanding intervention that is currently performed primarily in specialized centers with appropriate expertise. Its broader clinical implementation is still limited by maternal cell contamination, the lack of standardized protocols, and the scarcity of large prospective multicenter studies confirming its safety, reproducibility, and diagnostic reliability.

Future research should focus on optimizing procedural and laboratory methodologies, integrating advanced genomic technologies, and generating robust multicenter evidence regarding clinical outcomes and safety. In parallel, continued investigation of its therapeutic potential may open new avenues in fetal medicine. Although further validation is required before routine clinical implementation can be recommended, celocentesis should currently be regarded as an investigational technique rather than a standard prenatal diagnostic procedure. Its future clinical role will depend on the generation of robust multicenter evidence demonstrating safety, reproducibility, and clinical utility across diverse healthcare settings.

## Figures and Tables

**Table 1:** Summary of key clinical and methodological studies evaluating celocentesis for ultra-early prenatal diagnosis.

Study	Country/Center	Cases (*n*)	Indication	Gestational Age
Makrydimas et al., 2002 [68]	Greece, single center	108 celocentesis cases; 339 controls	Assessment of short-term safety of celocentesis	6–10 weeks
Makrydimas et al., 2004 [11]	Greece, single center	9	β-thalassemia (*n* = 6), sickle cell disease (*n* = 1), Marfan syndrome (*n* = 1), paternity testing (*n* = 2)	7–8 weeks
Jouannic et al., 2006 [28]	France, single center	14	Feasibility of prenatal diagnosis using coelomic fluid DNA	8–9 weeks
Giambona et al., 2016 [16]	Italy, single center	Not reported	Characterization of coelomic fluid cellular composition	7–9 weeks
Giambona et al., 2018 [18]	Italy, single center	489 (367 high-risk pregnancies; 122 elective terminations)	Hemoglobinopathies and fetal aneuploidy assessment	7–9 weeks
Makrydimas et al., 2020 [12]	Italy/Single center	402	Couples at risk for β-thalassemia or sickle cell disease	Median 8.6 weeks (range 6.9–9.9)
Giambona et al., 2022 [24]	Multiple studies	385	Development of laboratory workflow for prenatal diagnosis of monogenic diseases	7–10 weeks
Giambona et al., 2022 [14]	Italy, single center	4 pregnancies (3 couples)	Prenatal diagnosis of Cockayne syndrome	8 weeks
Vinciguerra et al., 2022 [52]	Italy, single center	1	Prenatal diagnosis for β-thalassemia with incidental chromosomal abnormality detection	8 weeks
Giambona et al., 2024 [15]	Italy, single center	5	Prenatal diagnosis of cystic fibrosis (*n* = 4) and cystic fibrosis with β-thalassemia co-inheritance (*n* = 1)	8 + 2 to 9 + 3 weeks

**Table 2:** Comparative analysis of celocentesis and conventional invasive prenatal diagnostic methods. Preimplantation genetic testing (PGT) is discussed separately because it represents a preconception reproductive strategy rather than a prenatal diagnostic procedure.

Feature	Celocentesis	Chorionic Villus Sampling (CVS)	Amniocentesis
Gestational age [11,91,92]	6–9 weeks	≥11 weeks	≥15 weeks
Biological source [22]	Coelomic fluid (embryo–fetal cells)	Placental trophoblast	Amniotic fluid (fetal cells)
Type of analysis [93,94]	Primarily molecular (monogenic ± targeted chromosomal analysis)	Molecular + cytogenetic + genomic	Molecular + cytogenetic + genomic
Diagnostic timing [95,96]	Ultra-early (embryonic period)	First trimester	Second trimester
Diagnostic accuracy [12,14]	93–99% (specialized centres)	>99%	>99%
Time to result [13]	Potentially rapid (PCR-based)	Moderate	Moderate
Procedure approach [12,96,97]	Transvaginal, ultrasound-guided	Transabdominal or transcervical	Transabdominal, ultrasound-guided
Maternal discomfort [98,99,100]	Minimal	Mild–moderate	Mild
Procedure-related miscarriage risk [12,101]	~2–2.3%	~0.5–1%	~0.1–0.3%
Placental or amniotic sac puncture [68,102]	No	Yes (placenta)	Yes (amniotic sac)
MCC [40,103]	High (requires active management)	Low	Low
Technical complexity [13,29]	High (operator- and laboratory-dependent)	Moderate	Moderate
Standardization [12,13]	Limited	Well established	Well established
Clinical maturity [13]	Experimental/specialized centers	Widely established	Widely established
Primary advantage [13,102,104]	Earliest possible diagnosis; potential therapeutic access	Early, well-validated diagnosis	Highly validated diagnostic method
Key limitation [13,42]	MCC, technical complexity, lack of standardization	Limited to ≥11 weeks gestation	Later diagnostic timing
Future potential [105,106]	In utero therapy, gene therapy delivery	Established diagnostic role with limited scope for earlier application	Established diagnostic role with limited scope for earlier application
Alternative reproductive strategy	PGT may be considered in IVF patients at high genetic risk before pregnancy is established		

**Table 3:** Potential future therapeutic applications of celocentesis and their current evidence base.

Application	Biological Rationale	Supporting Evidence	Potential Clinical Impact	Current Limitations
Hematopoietic stem cell transplantation [128,129,130]	Early fetal immune immaturity may allow engraftment	Stem cell viability demonstrated in coelomic fluid (preclinical primate models)	Treatment of hemoglobinopathies and immunodeficiencies	Limited clinical data, engraftment efficiency unknown
Gene therapy (in utero delivery) [131,132,133]	Early intervention may prevent disease expression	Feasibility of coelomic cavity access and fluid replacement demonstrated experimentally	Potential correction of monogenic disorders (e.g., CF, thalassemia)	Safety, delivery vectors, ethical concerns
Cell-based therapy [37,123,134]	Coelomic cavity as a delivery reservoir	Viability of transplanted cells in coelomic environment	Targeted early treatment strategies	Lack of human clinical trials
Early immunological tolerance induction [130,135,136,137]	Immature fetal immune system may reduce rejection	Theoretical and experimental support	Improved long-term outcomes of transplanted cells	Requires further validation
Drug or biologic delivery [11,23,138]	Direct access to embryonic compartment	Procedural feasibility established	Early targeted therapy	Pharmacokinetics unknown

## Data Availability

No new data were created or analyzed in this study.
